# Evaluating the Sources of Graphene’s Resistivity Using Differential Conductance

**DOI:** 10.1038/s41598-017-10367-1

**Published:** 2017-09-04

**Authors:** R. Somphonsane, H. Ramamoorthy, G. He, J. Nathawat, C.-P. Kwan, N. Arabchigavkani, Y.-H. Lee, J. Fransson, J. P. Bird

**Affiliations:** 1 Department of Physics, King Mongkut’s Institute of Technology Ladkrabang, Bangkok, 10520 Thailand; 2 Department of Electrical Engineering, University at Buffalo, the State University of New York, Buffalo, NY 14260-1900 USA; 3 Department of Physics, University at Buffalo, the State University of New York, Buffalo, NY 14260-1500 USA; 4 Department of Physics and Astronomy, Uppsala University, Box 516, SE-751 21 Uppsala, Sweden; 5 Graduate School of Advanced Integration Science, Chiba University, 1-33 Yayoi-cho, Inage-ku Chiba, 263-8522 Japan

## Abstract

We explore the contributions to the electrical resistance of monolayer and bilayer graphene, revealing transitions between different regimes of charge carrier scattering. In monolayer graphene at low densities, a nonmonotonic variation of the resistance is observed as a function of temperature. Such behaviour is consistent with the influence of scattering from screened Coulomb impurities. At higher densities, the resistance instead varies in a manner consistent with the influence of scattering from acoustic and optical phonons. The crossover from phonon-, to charged-impurity, limited conduction occurs once the concentration of gate-induced carriers is reduced below that of the residual carriers. In bilayer graphene, the resistance exhibits a monotonic decrease with increasing temperature for all densities, with the importance of short-range impurity scattering resulting in a “universal” density-independent (scaled) conductivity at high densities. At lower densities, the conductivity deviates from this universal curve, pointing to the importance of thermal activation of carriers out of charge puddles. These various assignments, in both systems, are made possible by an approach of “differential-conductance mapping”, which allows us to suppress quantum corrections to reveal the underlying mechanisms governing the resistivity.

## Introduction

In spite of the interest that graphene has attracted over the past decade^1^, the manner in which different scattering processes combine to determine its electrical resistance remains a topic of investigation. The reason for this may be traced to the ultrathin structure of this material, which allows its conduction to be influenced by a variety of different scattering mechanisms^1–23^. Phonon-mediated scattering, for example, can be generated by graphene’s intrinsic modes^2–9^, but can also be due to rippling^4, 10, 11^ or the underlying substrate^5, 12, 13^. Impurity scattering, on the other hand, is usually attributed to the presence of long-range charged impurities in the dielectric substrate^13–19^, and of short-range neutral defects in the graphene itself^15, 16, 18, 20, 22^. Collectively, these mechanisms may combine to yield complicated variations of the resistance as a function of both temperature (*T*) and carrier density (*n* & *p* for electrons and holes, respectively). A problematic task in experiment is that of separating out the different contributions, a job that is made all the more challenging at low temperatures by the emergence of quantum corrections^24–29^. Most notable of these are those arising from weak (anti) localization^24–27^ and electron-electron scattering^28, 29^, although Kondo physics has also been invoked to account for the behaviour observed in this regime^30–34^.

In conventional semiconductors near room temperature, and with modest levels of doping, phonon scattering is typically the dominant process limiting carrier mobility (*µ*). The situation in graphene is more complicated, however, not only for the reasons identified above. Electrons and holes reside on a two-dimensional Dirac cone, placing significant phase-space restrictions on electron-phonon scattering^7, 35–37^. The large (>160 meV) energy of the graphene optical modes is additionally understood to suppress their importance, and the overall message is that phonon scattering is weak^35–37^. Room temperature mobility as large as ∼20,000 cm^2^/Vs is therefore expected for graphene on SiO_2_^17, 19^, while experiment has demonstrated values an order of magnitude larger for suspended layers^4, 6^. In many other experiments (performed with graphene on SiO_2_), however, observed mobility values may be smaller than 1000 cm^2^/Vs^38, 39^, suggesting that the contribution to the resistivity from impurity scattering can be much more important than in conventional semiconductors.

In this article, we study the resistance of graphene/SiO_2_ transistors over a wide range of temperature (3–300 K), and for densities spanning the electron and hole branches of the Dirac cone. Due to their different energy dispersions, the importance of Coulomb and short-range impurities is expected to be very different in monolayer and bilayer graphene^22^. We therefore perform a comparative study of transistors fabricated with these materials. While prior work has largely focused on the resistance variations exhibited by graphene under linear transport^2–23^, far fewer studies^40–44^ have applied differential-conductance measurements to this problem. Here we use measurements of differential-conductance, performed as a function of temperature, density and drain bias, to identify the different contributions to graphene’s resistance. Crucially, this allows us to identify resistivity contributions, free of the influence of quantum corrections. Our findings in both the monolayer and bilayer systems are in good agreement with the theories^1, 20, 22, 23, 45^ proposed by Das Sarma and his colleagues.

## Results

### Linear Conductance of Monolayer and Bilayer Graphene

**Figure 1:**
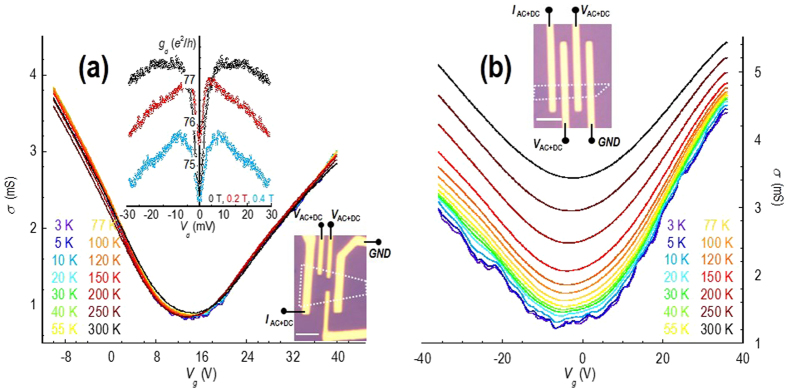
Variation of linear conductivity with gate voltage (*V*_*g*_) for the monolayer (panel (a)) and bilayer (panel (b)) devices at various temperatures in the range of 3–300 K. Optical micrographs of the two devices are shown in the corresponding insets, in both of which the outline of the graphene flake is indicated by the white dotted line and current and voltage probes are indicated. The scale bar denotes a distance of 4 μm. The upper inset to panel (a) shows the differential conductance of the monolayer device as a function of drain bias at 3 K, and for three different values of magnetic field (indicated).

We begin our discussion by first of all addressing the temperature dependence of graphene’s linear transport, a problem that has previously been widely investigated^2–23^. To make a direct connection to these earlier works, in Fig. 1 we plot the variation of the conductivity, as a function of gate-voltage (*V*_*g*_) and temperature. In Fig. 1a we show that the conductivity of monolayer graphene is only weakly dependent on temperature, while in Fig. 1b the bilayer shows a much stronger variation. The weak temperature dependence observed in the monolayer indicates that the dominant source of resistivity in this material, at all densities, is long-range Coulomb disorder. According to ref. 4, the contribution to the resistivity due to this mechanism should not depend on temperature below 300 K. This is in contrast with our results for the bilayer system, which shows a pronounced increase in overall conductivity with increasing temperature. The reason for this much stronger temperature dependence may be attributed to two factors, the first of which is that the thermal carrier concentrations increases much more strongly with increasing temperature in the bilayer. Secondly, due to the very different nature of screening in the two materials, scattering from short range defects, rather than Coulomb impurities, is expected to dominate in the bilayer, and is characterized by a strongly temperature-dependent nature^20, 22^.

As a final comment, we note that Fig. 1 shows that, away from the Dirac point, the conductivity in both devices varies linearly with gate voltage (and so with *n* or *p*). While these variations might appear to suggest a common origin, in both materials, this, in fact, is not the case. Rather, the observed behaviour is completely consistent with what one expects when scattering in the monolayer is dominated by screened Coulomb impurities^4, 17–22^, and that in the bilayer is governed by unscreened short-range disorder^22^.

### Differential-Conductance Mapping of Monolayer and Bilayer Graphene

**Figure 2:**
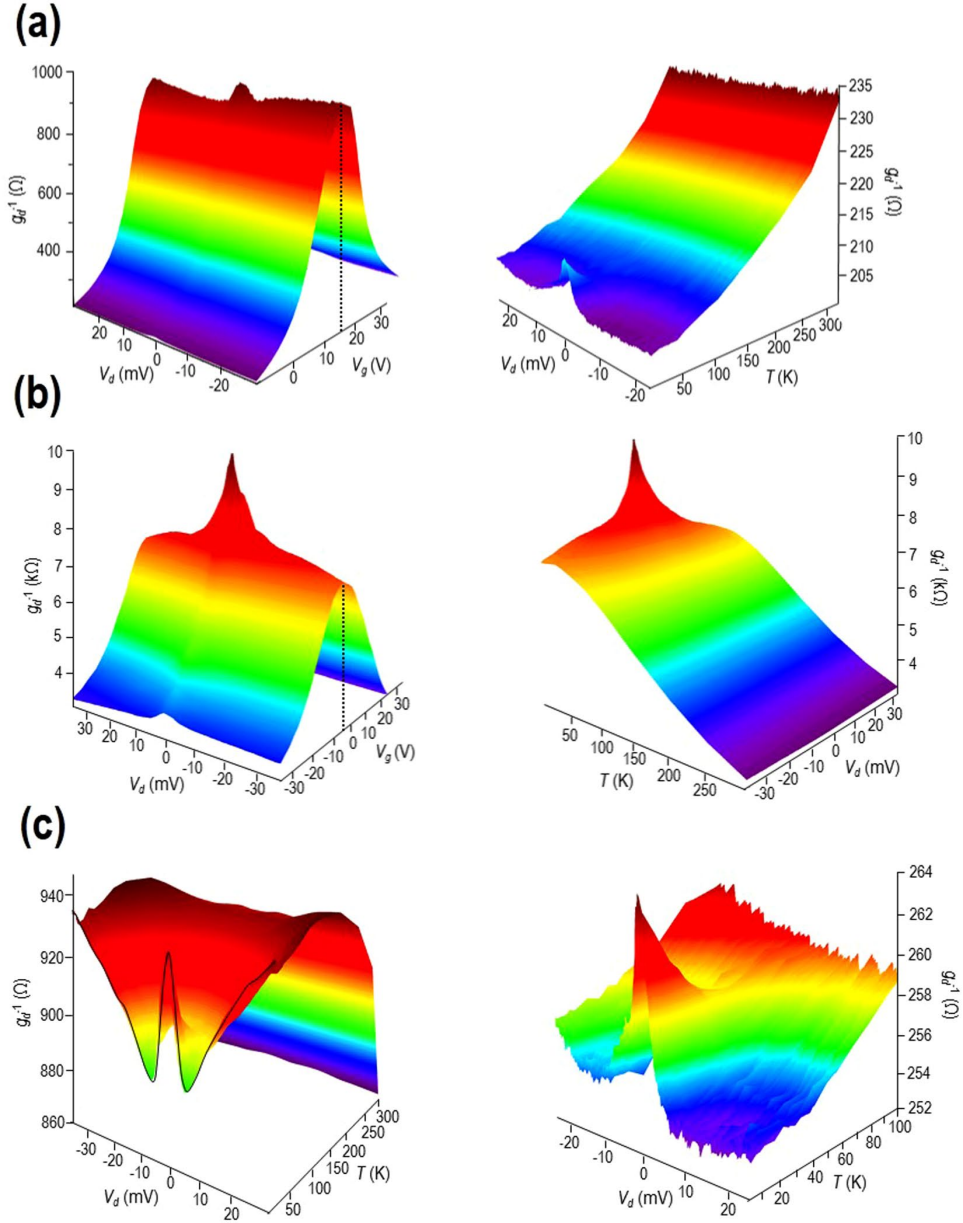
Differential conductance mapping of the monolayer (panel (a)) and bilayer (panel (b)) devices. The contour shown on the left-hand side of each of these two panels plots the full evolution of the differential resistance (*g*_*d*_^−1^) as a function of *V*_*d*_ and *V*_*g*_ at *T* = 3 K. The dotted lines indicate the position of the Dirac point. The contours shown on the right-hand side of each panel plot the dependence of differential resistance (*g*_*d*_^−1^ (*V*_*d*_)) for fixed gate voltage and as a function of temperature. For the monolayer device the gate voltage was kept at −10 V corresponding to the induced hole density (*n*_*g*_) of 1.7 × 10^12^ cm^−2^, while for the bilayer device the gate voltage was −6 V and the induced hole density was 1.1 × 10^11^ cm^−2^. (Note that these values are not the total hole concentrations, *p*, which were also determined by *n* ^*^ and *n*_*th*_). Panel (c) show the variation of differential resistance (*g*_*d*_^−1^) as a function of drain bias and temperature in the monolayer. The contour on the left is obtained for a gate voltage close to the Dirac point (*V*_*g*_ = 14 V), while on the right is obtained away from the same point (*V*_*g*_ = −10 V).

While linear transport provides a useful tool to reveal the role of different scattering mechanisms, for a more complete picture, offering energy resolution, it is necessary to make use of nonequilibrium studies. It is this approach that we apply here, where we perform differential-conductance mapping of graphene, as a function of drain bias, gate voltage, and temperature. The value of this approach is highlighted in Fig. 2, where we show contour plots of differential conductance (*g*_*d*_) as a function of these parameters. (More precisely, the plots show differential resistance *g*_*d*_^−1^, since it is more easy to resolve the different features of the resulting contours in this case.) Fig. 2a,b are for monolayer and bilayer graphene, respectively. While their left panels plot (at the lowest temperature of 3 K) the variation of *g*_*d*_^−1^ as a function of *V*_*d*_ and *V*_*g*_, the right-hand panels plot the differential resistance at fixed density (see figure caption) and as a function of temperature. The Dirac point is identified as a pronounced peak at *V*_*g*_ = 14 and *V*_*g*_ = −5 V, for monolayer and bilayer graphene, respectively, as indicated by the dotted lines in the left-hand panels. The main features of these contours are: (1) The zero-bias peak in differential resistance that is strongly suppressed with increasing temperature, washing out around 40–50 K; (2) The pronounced temperature-dependent variations of *g*_*d*_^−1^, away from the zero-bias peak, which appear to be of opposite sign for the monolayer and bilayer systems.

### Zero-Bias Resistance Peak Due to Quantum Corrections

The zero-bias peak (Fig. 2) in the low-temperature differential resistance (*g*_*d*_^−1^) is consistent with the influence of quantum corrections, most notably weak localization and electron interactions^46^. As can be seen in the data of Fig. 2, the peak washes out around 40 K or so (this can be seen more clearly in the right panel of Fig. 2c), consistent with its origins in a quantum-coherent effect^46^. Furthermore, in the upper inset to Fig. 1a, we plot the differential conductance of the monolayer device at three magnetic fields. While the largest of these (0.4 T) should be sufficient to fully quench weak localization^28^, the amplitude of the zero bias peak is only reduced by ∼50% compared to its size at zero magnetic field. This indicates that this feature arises from a combination of weak localization and electron interactions, both of which become increasingly important as the temperature is lowered and carrier coherence is enhanced^46^ (see Section S5 of the Supplementary Information).

**Figure 3:**
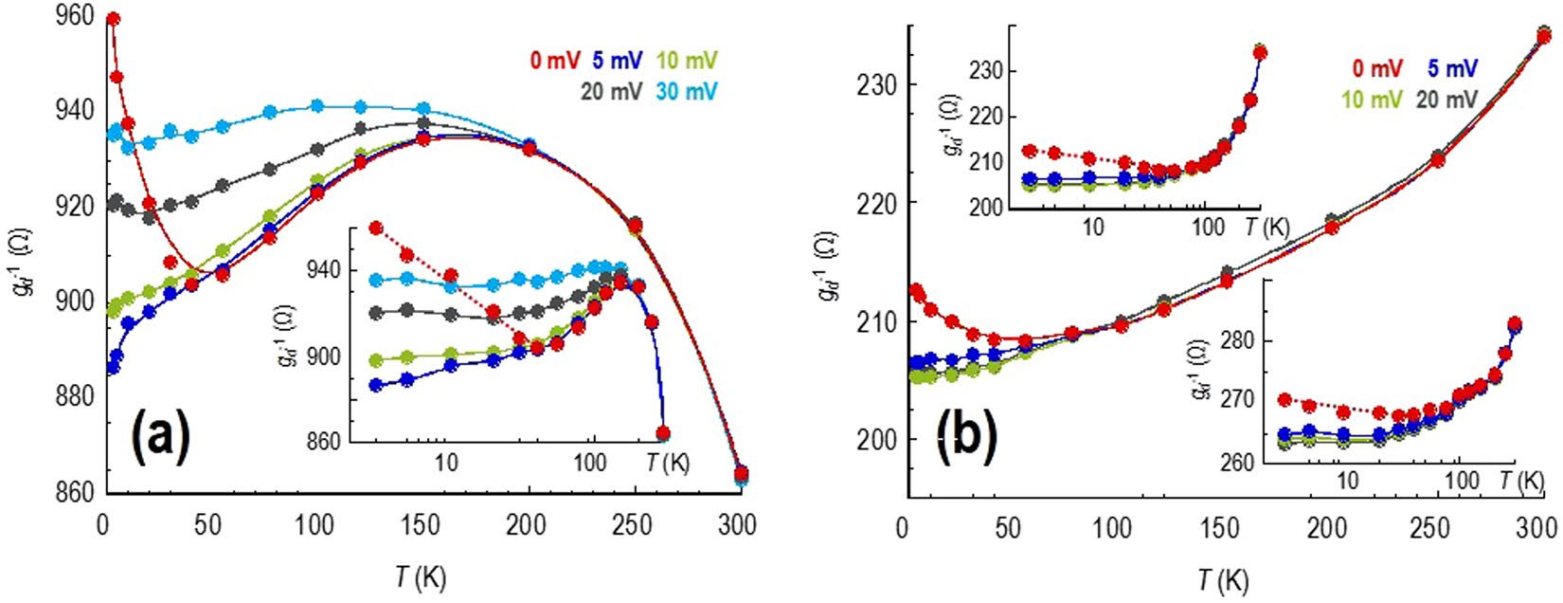
The main panels of (a) and (b) show the temperature dependent variation of the differential resistance (*g*_*d*_^−1^(*T*)) of the monolayer device, measured using various fixed values of the drain bias (indicated). Panel (a) is for a hole density close to the Dirac point (*V*_*g*_ = 14 V) and in the inset its data are re-plotted on a semi-log scale, highlighting the presence of a logarithmically-increasing contribution to the resistance at low temperatures and zero bias. Panel (b) is for a higher hole density (at *V*_*g*_ = −10 V) and its upper inset re-plots the same data of the main panel on a semi-log scale, once again highlighting the presence of a logarithmically-increasing contribution to the resistance at low temperatures and zero bias. In the lower inset we show a similar plot to the upper inset, but in this case obtained on the electron side of the Dirac curve (*V*_*g*_ = +40 V).

**Figure 4:**
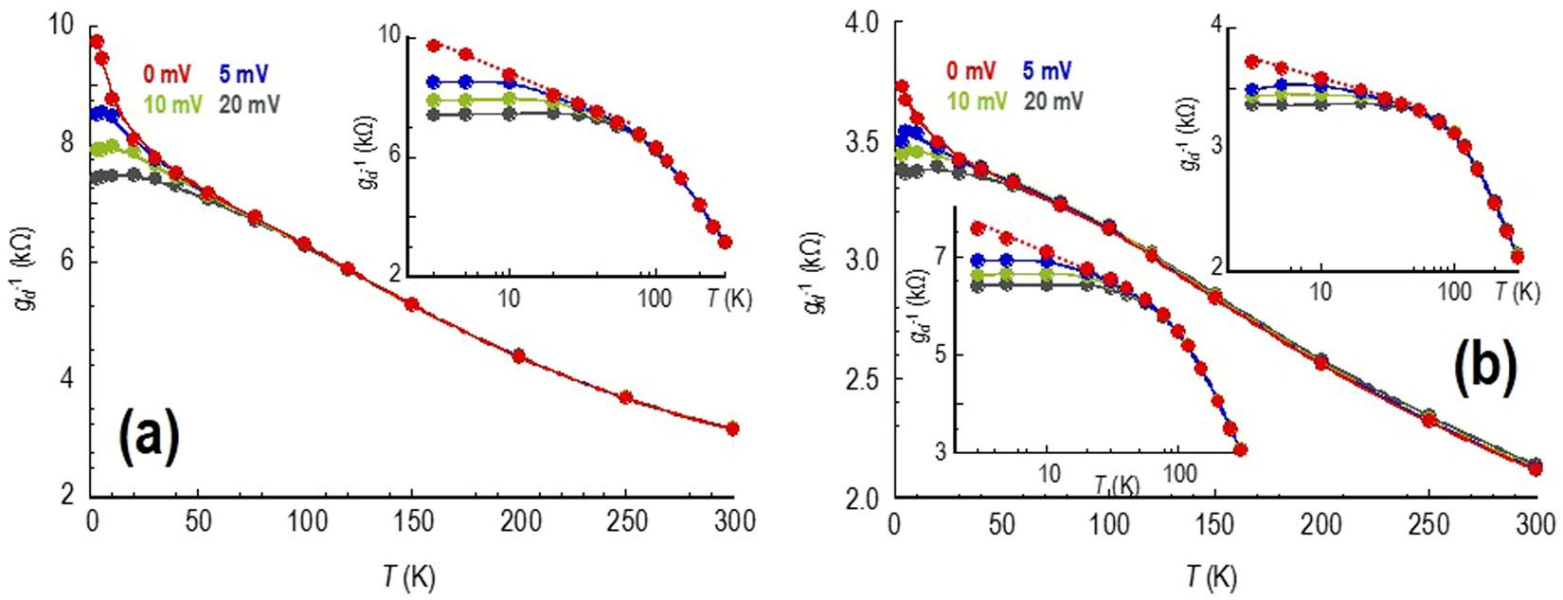
The main panels of (a) and (b) show the temperature dependent variation of the differential resistance (*g*_*d*_^−1^(*T*)) of the bilayer device, measured using various fixed values of the drain bias (indicated). In panel (a) the gate voltage is −6 V, while in panel (b) it is −36 V. The upper insets to panels (a) and (b) are plotted on a semi-log scale, highlighting the presence of a logarithmically-increasing contribution to the resistance at low temperatures and zero bias. The lower inset to panel (b) plots the corresponding resistance variation measured for a gate voltage of 6 V.

To observe how the resistance is influenced by semiclassical contributions such as phonon, and charged-impurity, scattering, it is necessary to suppress the quantum corrections by suitable means. We achieve this here by measuring the variation of resistance in the presence of non-zero DC bias, as we demonstrate in Figs 3 & 4. The essential observation here is that, for *V*_*d*_ = 0, the resistance of both monolayer (Fig. 3a,b) and bilayer (Fig. 4a,b) graphene increases as a logarithmic function of decreasing temperature below ∼40 K. This behavior is well known^46^ for quantum corrections and can be quenched by measuring the temperature dependence of the resistance under non-zero DC bias. In Fig. 3a, for example, the zero-bias resistance measured (near the Dirac point) in monolayer graphene shows its logarithmic upturn below 50 K (also see the inset to the main panel). With a DC bias of 10 mV, however, the logarithmic upturn is completely suppressed and the resistance decreases continuously down to 3 K. A similar suppression is apparent for electron and hole densities far from the Dirac point (Fig. 3b, main panel and insets), as well as in bilayer graphene (Fig. 4a,b), and should result from the suppression of carrier phase coherence as the DC bias is increased. The essential point for the study here is that, by suppressing the influence of the quantum corrections, we are able to investigate the underlying sources of graphene’s resistance.

### Resistance Variations in Monolayer and Bilayer Graphene

Having described how the quantum corrections may be suppressed in our studies, in this section we focus in more detail on the resistance variations exhibited by the monolayer (Fig. 3) and bilayer (Fig. 4) devices. The most striking feature of Fig. 3a, in which we plot the results of measurements obtained with the Fermi level close to the Dirac point, is a nonmonotonic variation of the resistance (also see the contour in the left panel of Fig. 2c). Even with the quantum corrections suppressed at *V*_*d*_ = 10 mV, the resistance still exhibits this nonmonotonic character; increasing first as the temperature is lowered below 300 K, before crossing over to a metallic-like variation for which the resistance decreases below 150–200 K. A similar variation is apparent for larger voltages (*V*_*d*_ > 10 mV) but the resistance no longer drops at low temperatures, instead showing a tendency to saturation. This is presumably due to self-heating of the graphene carriers under the strongly nonequilibrium voltage^47^; the voltage of 20 mV, for example, corresponds to an equivalent temperature of 200 K, consistent with the temperature below which the resistance saturates in Fig. 3a.

In Fig. 3b we show the corresponding variations of the resistance, exhibited by the monolayer system once the hole concentration has been adjusted to *n*_*g*_ = 1.7 × 10^12^ cm^−2^. With the quantum corrections suppressed by the application of the DC bias, the resistance at higher temperatures is characterized by a monotonically-increasing (metallic) variation. Similar behaviour is apparent, also, in the lower inset to Fig. 3b, where we plot the corresponding results obtained for an electron density of 1.9 × 10^12^ cm^−2^.

While we have focused thus far on the behaviour exhibited by monolayer graphene, in panels (a) & (b) of Fig. 4 we plot a series of graphs that are the bilayer counterparts of Fig. 3a,b, respectively. Comparing the monolayer and bilayer devices, we immediately note that (with quantum corrections suppressed) the latter exhibits a much simpler, monotonic, decrease of resistance with increasing temperature. This insulator-like behaviour is observed both close to (Fig. 4a), and away from (Fig. 4b), the Dirac point. Similar results, obtained for another bilayer device, are presented in Section S6 of the Supplementary Information. Critically, the continuous increase of conductance with increasing temperature cannot be explained within any model of phonon-limited conduction in this material.

## Discussion

Before discussing the implications of our experimental results it will be helpful to first of all summarize some of the theoretical predictions regarding the sources of resistivity in graphene. Phonons should always give rise to an increasing (metallic) resistivity with increasing temperature, with acoustic phonons providing a linear-in-*T* contribution over the range of interest here^3, 5, 7^. With regards to scattering from optical phonons, the large energies associated with these modes is often thought to render them unimportant at or below room temperature. However, recent first-principles calculations^8, 9^ based on density-functional theory suggest that, above ∼200 K, optical-phonon scattering may actually become stronger than that due to the acoustic modes, resulting in a metallic resistivity that shows a more rapid increase than linear-in-*T*.

Impurity scattering in graphene is typically attributed to Coulombic impurities in the SiO_2_ substrate and to neutral defects in the graphene layer. With regards to charged impurities, the influence of these is strongly dependent on the screening generated by carriers in the graphene. Screening enters the matrix element for the scattering process via the dielectric function, which in turn depends upon the polarizability of the material. The key point here is that the dependence of this function on temperature and scattering wavevector is very different in monolayer and bilayer graphene, resulting in very distinct screening properties in these materials^20, 22^. Most notably, in monolayer graphene the polarizability shows a nonmonotonic dependence on temperature, which should give rise to a similarly nonmonotonic variation of the resistivity. Hwang and Das Sarma^20^ have calculated this behaviour analytically, accounting for the influence of thermal carriers via usual Fermi-Dirac statistics, obtaining asymptotic expressions for the monolayer conductivity:

1 $$\sigma (T)\approx {\sigma }_{0}[1-\frac{2{\pi }^{2}{r}_{s}}{3}\frac{{I}_{1}}{{I}_{0}}{(\frac{T}{{T}_{F}})}^{2}]\,(T\ll {T}_{F})\,\&$$

2 $$\sigma (T)\approx {\sigma }_{0}[\frac{16{I}_{0}}{\pi }{(4(\mathrm{ln}2){r}_{s})}^{2}{(\frac{T}{{T}_{F}})}^{2}]\,(T\gg {T}_{F}),$$

where *σ*_0_ = *σ*(*T* = 0), *T*_*F*_ is the Fermi temperature, *r*_*s*_ is the Wigner-Seitz radius and:

3 $${I}_{n}={\int }_{0}^{1}\frac{{x}^{2}\sqrt{1-{x}^{2}}}{{(x+2{r}_{s})}^{2+n}}.$$

When the explicit temperature dependence of the finite energy averaging of the scattering time is considered, and by taking *r*_*s*_ = 0.88 (for graphene on SiO_2_), equation (1) is slightly modified to^20^:

4 $$\sigma (T)={\sigma }_{0}[1-\frac{{\pi }^{2}}{3}0.21{(\frac{T}{{T}_{F}})}^{2}]\,.$$

Das Sarma *et al*. have also considered^22^ the influence of temperature-dependent screening in bilayer graphene, and predict a metallic resistivity contribution at low temperatures (*T*/*T*_*F*_ ≪ 1). A critical difference with the monolayer, however, is that scattering from short-range impurities is expected to be much more important in bilayer material. The short-range scatterers give rise to a strong insulating temperature dependence of the resistivity in this system, which can overwhelm any signature from charged impurities. (This is to be contrasted with monolayer graphene, in which the influence of short-ranged scatterers is only expected^20, 22^ to be significant at very-high densities – ∼10^13^ cm^−2^ – once screening of the Coulomb impurities become fully effective.) While the resistivity correction arising from short-range scatterers can only be evaluated numerically, the authors of ref. 22. showed that this mechanism should give rise to an insulating variation of the scaled conductivity (*σ*(*T*/*T*_*F*_)/*σ*_0_) that is *independent* of density.

In later work^23, 45^ by the Maryland group, the authors addressed explicitly the thermal activation of carriers out of localized puddles arising from the disorder background. Here the authors discussed the puddles as giving rise to a “two-component” model of transport; the first involving usual diffusive transport in the graphene layer, while the second involves the thermal activation of carriers out of the puddles. The activated transport is predicted^23, 45^ to give rise to an insulating conductivity near the Dirac point in both monolayer and bilayer graphene systems (equations (5) & (6), respectively, valid when *k*_*B*_ *T* < *s*):

5 $$\sigma (T)={\sigma }_{0}[1+\sqrt{\frac{2}{\pi }}\frac{{k}_{B}T}{s}+\frac{{\pi }^{2}}{3}{(\frac{{k}_{B}T}{s})}^{2}],$$

6 $$\sigma (T)={\sigma }_{0}[1+\sqrt{\frac{2}{\pi }}\frac{{k}_{B}T}{s}+\frac{{\pi }^{2}}{6}{(\frac{{k}_{B}T}{s})}^{2}],$$

where *s* is the strength of the potential fluctuations in graphene and is typically expected to be in the range of 10–100 meV. The thermal activation is expected to be most significant in the bilayer system, in which the extent of puddle formation should be much stronger, leading to larger characteristic values of *s* ^23, 45^. Nonetheless, evidence for the activation has been found in disordered, CVD-grown, monolayer graphene^48^. Here, the competition between thermal activation, temperature-dependent screening, and phonon scattering was found to result in a nonmonotonic variation of the conductivity, in the crossover regime between insulating and metallic states.

The presence of puddling in typical experiments usually obscures the intrinsic variations of the conductivity that are expected for graphene. Here, the conductivity near its Dirac point is predicted to follow the semiclassical transport theory proposed in ref. 49. According to this theory, which accounts for the influence of thermal excitation, screening, and scattering, the conductivity should be insulating in character, and follow a power-law variation as a function of temperature. While this behaviour is not observed in most experiments, such as those performed here where the graphene is supported on an SiO_2_ substrate, there is evidence to suggest that it can be observed at sufficiently low densities in clean, suspended material^6, 50–53^.

**Figure 5:**
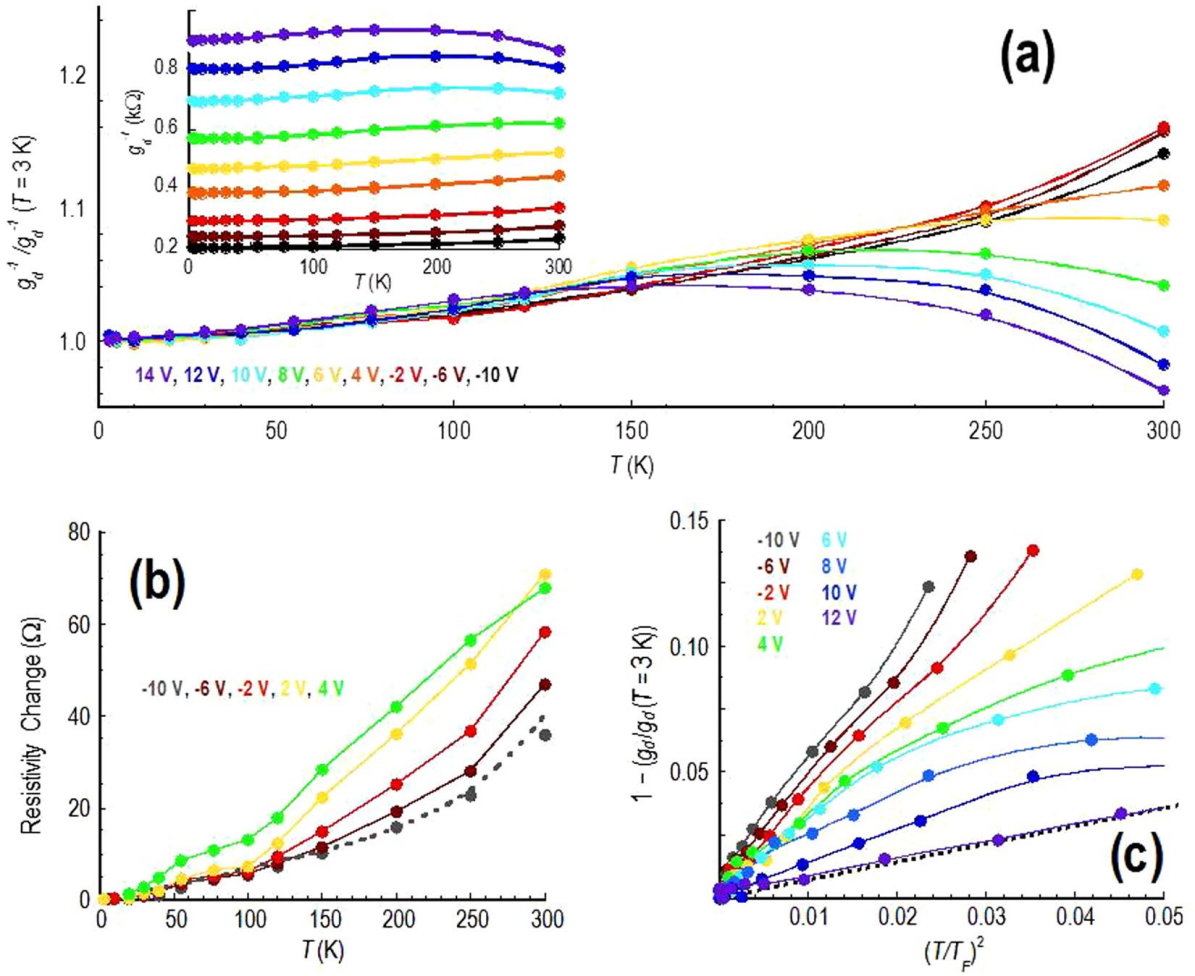
Temperature-dependent variation of the differential resistance (*g*_*d*_^−1^(*T*), *V*_*d*_ = 10 mV) of the monolayer device. (**a**) The inset shows the variation of differential resistance at several gate voltages (identified in the main panel), while in the main panel these data are re-plotted by normalizing them relative to the low-temperature (*T* = 3 K) value. A crossover from monotonic to nonmonotonic behaviour occurs as the Dirac point (at *V*_*g*_ = 14 V) is approached from the hole band. (**b**) The temperature-dependent change of resistivity (measured relative to the value at 3 K, *V*_*d*_ = 10 mV, gate voltages indicated) for selected data from panel (a). The dotted line in the figure is an extrapolation of data presented in the theoretical study of refs 8 & 9. (**c**) The data of panel (a) are replotted to reveal the connection to the predictions of equation (4). The dotted line in the figure is the straight-line variation expected from this equation with *r*_*s*_ = 0.88.

With this understanding, we now consider the implications of our experimental results in the light of the predictions above. We begin, in Fig. 5, by performing an analysis of the resistance variations observed in monolayer graphene, with a non-zero DC bias (*V*_*d*_ = 10 mV) applied to suppress its quantum-corrections. In the inset to Fig. 5a, we plot resistance as a function of temperature for a number of gate voltages on the hole side of the Dirac curve (*V*_*g*_ ≤ 14 V). These data are re-plotted in the main panel of the figure, by rescaling them relative to their low-temperature resistance (at 3 K). Starting from a gate voltage of −10 V, the Fermi level lies well within the valence band (see Fig. 1a) and the resistance exhibits a metallic variation over the entire temperature range. As the gate voltage is increased towards the Dirac point (at *V*_*g*_ = 14 V), however, the resistance develops a nonmonotonic character, the onset of which appears at progressively lower temperatures for larger gate voltages. For *V*_*g*_ = 6 V, for example, the crossover to an insulating resistance occurs around 250–300 K; by the time the Dirac point is reached, however, the onset has shifted down to ∼150 K.

The data of Fig. 5a support a scenario involving a crossover from phonon-dominated conduction, when the Fermi level lies far from the Dirac point, to one in which transport is governed by scattering from screened Coulomb impurities when the Fermi level is near to this special point. In Section S4 of the Supplementary Information, we include a table relating the different gate voltages to the corresponding electrostatically-induced concentrations (*n*_*g*_) that they imply. From this table, we see that the data in this figure span a wide range of carrier density, from *n*_*g*_ ∼ 2 × 10^12^ cm^−2^ in the highest case to almost *n*_*g*_ ∼ 0 near the Dirac point (at *V*_*g*_ = 14 V). In Fig. 5b, we then address the origins of the resistance variations observed well away from the Dirac point, by plotting the temperature-dependent change of resistivity (relative to its value at 3 K). At these hole concentrations (>7 × 10^11^ cm^−2^), the gate-induced carrier density is larger than that due to both residual impurities (n*) and thermal carriers (*n*_*th*_), even at room temperature. (For a further discussion of this point see Section S4 of the Supplementary Information, where we also show how consistent estimates for the residual impurity density can be obtained from a Raman analysis – see Fig. S1 – or from the features of the Dirac curve – see Fig. S4). In this limit we find that the resistance shows purely metallic behaviour, increasing monotonically with increasing temperature. At the same time, as *n*_*g*_ is increased through variation of the gate bias in this figure, the overall resistance level systematically decreases. The dotted line included in the figure represents an extrapolation of the data of Park *et al*., who have calculated the phonon-limited resistivity for graphene by first-principles methods^8, 9^. Their calculations employ both density-functional theory, and density-functional perturbation theory, to compute all electronic and phononic properties, including the influence of resultant electron-phonon coupling. The dotted line in Fig. 5b clearly agrees well with our experimental data for the gate voltage of −10 V (corresponding to a hole density of *n*_*g*_ = 1.7 × 10^12^ cm^−2^). According to Park *et al*., the phonon-limited resistivity is governed by acoustic modes at low temperatures, whereas at temperatures above ∼150 K the role of optical phonons becomes more important. It is this crossover in scattering that we attribute to the rapid upturn of the resistivity seen in experiment beyond 150 K. From this analysis we therefore conclude that, when the concentration is dominated by the influence of gate-induced carriers, the resistivity of graphene is essentially determined by phonon scattering. This situation changes when the gate-induced concentration of carriers decreases, however, as we now discuss.

As the gate voltage is increased beyond 6 V in Fig. 5a, the hole concentration drops below *n*_*g*_ = 5 × 10^11^ cm^−2^ and it is clear that the scaled resistance deviates from a purely metallic variation that is the signature of electron-phonon scattering and develops, instead, a nonmonotonic dependence on temperature. The onset of this behaviour initially occurs near room temperature, but decreases to ∼150 K as *n*_*g*_ → 0. A clue as to the origins of this nonmonotonicity, is provided again in Section S4 of the Supplementary Information. This figure shows that, for *n*_*g*_ < 5 × 10^11^ cm^−2^, one enters a new regime where the full carrier concentration is strongly modified by the contribution from charged-impurity induced residual carriers. On the basis of these observations, we attribute the nonmonotonic variations seen in Fig. 5a to the role of scattering from screened Coulomb impurities, as predicted by Hwang and Das Sarma^20^. To establish this point, in Fig. 5c we have rescaled the data from Fig. 5a to reveal a connection to equation (4). The graph focuses on the low-temperature limit (*T* << *T*_*F*_), where the prediction of equation (4) is expected to be valid^20^, and the dotted line in the figure represents the universal variation this describes (for *r*_*s*_ = 0.88). It is clear that good agreement with this line is obtained for the lowest density studied, where the gate-induced concentration (*n*_*g*_ = 1.4 × 10^11^ cm^−2^) is in fact smaller than that (∼2 × 10^11^ cm^−2^) due to the Coulomb impurities. As the gate voltage is used to produce heavier hole doping, however, the conductance rises rapidly above the dotted line in Fig. 5c. The suggestion of these results is therefore as follows. At the lowest density that we study (*n*_*g*_ < 5 × 10^11^ cm^−2^), the resistivity data closely follow the universal variation expected^20^ for scattering from screened Coulomb impurities, indicating that this mechanism dominates over phonon scattering in this limit. As the density is increased, however, the data in Fig. 5c rise above the universal curve, indicating that an additional mechanism is contributing significantly to the resistivity. This mechanism is presumably due to phonon scattering, which grows to eventually dominate the measured resistance once the density becomes sufficiently large (*n*_*g*_ > 10^12^ cm^−2^).

While in the above discussion we have characterized our results in terms of “metallic” and “insulating” resistivity variations, it must be emphasized that this has nothing to do with a metal-insulator transition. Indeed, even with the nonmonotonic character of the resistance manifested most strongly near the Dirac point, the minimum conductivity achieved in the monolayer is ∼20 *e* ^2^/*h* per square (see Sections S1 & S3 of the Supplementary Information). The graphene is therefore metallic at all densities studied, and the observation of “metal-like” and “insulator-like” resistance variations simply reflects the competition between the different scattering mechanisms noted above.

**Figure 6:**
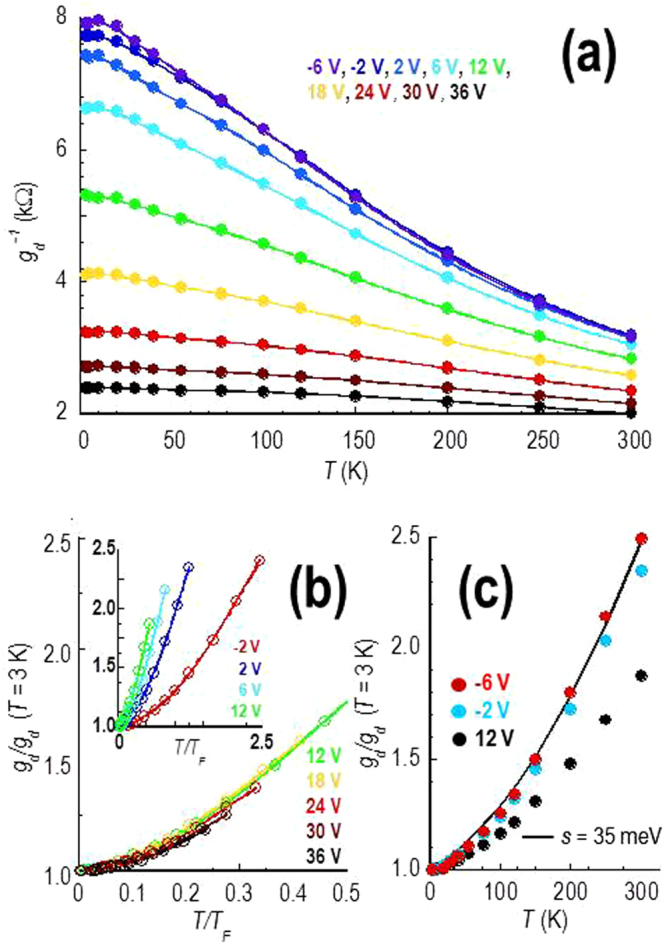
Temperature-dependent variation of the differential resistance (*g*_*d*_^−1^(*T*), *V*_*d*_ = 10 mV) of the bilayer device. (**a**) *g*_*d*_^−1^ as a function of *T* for several gate voltages on the electron side of the Dirac curve. (**b**) Variation of the rescaled differential *conductance* (*g*_*d*_/*g*_*d*_(*T* = 3 K)) as a function of *T*/*T*_*F*_ for several gate voltages on the electron side of the Dirac curve. Inset: Same as in main panel but for a wider range of *V*_*g*_. (**c**) Selected data from the inset to Fig. 6b are replotted to compare with the theoretical prediction (solid line) of the two-component transport model of refs 23, 45.

Turning to the variations exhibited by bilayer graphene, these are somewhat easier to analyse since they show no evidence of any metallic variation that would provide a signature of electron-phonon scattering. The first point we note from Fig. 1 is that the overall conductivity in bilayer material is much lower than that in the monolayer, a difference that is commonly observed in experiment and which is attributed to differences in the density of states and screening properties of these materials^54^. The different density of states is also responsible^54^ for the increased importance of thermal carriers in the bilayer system, relative to the monolayer (see Section S3 of the Supplementary Information). As we indicate in Fig. 6a, where we plot the variation of resistance with temperature for electrons, the resistance of this material therefore shows insulating variations for all temperatures and densities. This appears consistent with the predictions for scattering from short-range defects in bilayer material^22^. As described above, the signature of such scattering in the bilayer should be a density-independent variation of the scaled conductivity (*σ*(*T*/*T*_*F*_)/*σ*_0_). This behaviour can be clearly seen in the main panel of Fig. 6b, where we plot rescaled differential conductance as a function of *T*/*T*_*F*_ for a number of electron densities in the range of *n*_*g*_ = 1.2 – 2.9 × 10^12^ cm^−2^ (see the table included in Section S4 of the Supplementary Information for the correspondence of *V*_*g*_ to *n*_*g*_ for the bilayer). Over this density range, where the contribution from gate-induced carriers dominates the total electron or hole density (see Section S4 of the Supplementary Information), the data clearly fall on a common curve expected for short-range scatterers^22^. This is obviously very different to the behaviour discussed for monolayer graphene, in which the resistivity at higher densities was instead dominated by electron-phonon scattering (Fig. 5b). This may be understood in terms of the different screening properties of monolayer and bilayer graphene^22^. In the inset to Fig. 6b, we extend our analysis by including data for lower electron densities (*n*_*g*_ = 0.1–1.2 × 10^12^ cm^−2^) than those in the main panel. Here it is clear that the data progressively deviate from the density-independent curve, as *n*_*g*_ is reduced to 1.0 × 10^11^ cm^−2^. Since this concentration corresponds to the situation where *n*_*g*_ < *n* ^*^, one might expect that the transport should be increasingly limited by puddling behaviour associated with charged background impurities. Consistent with this, in this regime, we find that the temperature dependence of the conductance is well described by the prediction of equation (6) for two-component transport^23, 45^. This is shown in Fig. 6c, where we are able to fit the data near the Dirac point with a reasonable^23, 45^ estimate (*s* = 35 meV) for the potential-fluctuation strength.

In conclusion, in this article we have explored the contributions to the resistance of monolayer and bilayer graphene transistors. Our studies have revealed transitions between different regimes of scattering, as a function of density, temperature, and of the material system, the details of which appear consistent with the predictions of theory^1, 20, 22, 23, 45^. In monolayer graphene, the temperature dependence of the resistivity near the Dirac point appears to arise predominately from scattering from screened Coulomb impurities, whereas at higher densities it shows signatures of acoustic and optical phonons. In bilayer graphene, short-range impurities dominate the temperature-dependence of the resistivity for nearly all densities. The exception to this behaviour arises near the Dirac point, where thermal activation out of charge puddles becomes significant. A key approach here has been the application of differential-conductance measurements to suppress the influence of quantum corrections, which has allowed us to identify the various transport regimes relevant to these materials. This approach is expected to be broadly applicable to other low-dimensional materials, and should find application, for example, in the study of transition-metal dichalcogenides and topological insulators.

## Methods

Graphene devices were fabricated by exfoliating Kish graphite onto a doped Si substrate with a 300-nm SiO_2_ cap layer, and an underlying, heavily-doped, Si layer that served as a back gate^47^. Individual graphene flakes were then contacted with Cr/Au (3-/50-nm) electrodes, defined by electron-beam lithography and lift-off. Layer identification was achieved through a combination of optical microscopy and Raman imaging (see Section S2 of the Supplementary Information). Various devices were fabricated, and characterized electrically, and in the Supplementary Information we show how these exhibited similar and consistent characteristics, independent of the contact configuration used (see Section S1 of the Supplementary Information). In this report, we focus on a detailed study of the differential conductance exhibited by two representative devices, one monolayer and the other bilayer. Optical micrographs of these devices can be seen in the insets to Fig. 1. The various measurements reported here were performed in a four-terminal geometry, thereby eliminating the influence of any contact resistance. In the micrographs of Fig. 1 we indicate how this was achieved, by denoting the two contacts that were used to apply an external (AC and/or DC) voltage(s), and the two internal probes that were then used to measure the resulting voltage drop (*v*) across a region that then served as our channel. Two different kinds of measurement were made in this study; linear conductance (or resistance) was determined by a low-frequency (13-Hz) AC technique, using a small AC signal (*v*_*d*_ = 100-μV RMS) as the externally-applied voltage. For measurements of differential conductance (*g*_*d*_), a DC voltage (*V*_*d*_) of varying amplitude was applied on top of this AC component, allowing us to perform differential-conductance spectroscopy as a function of both *V*_*d*_ and gate voltage (*V*_*g*_). An important point that must be emphasized here is that, in all figures and associated text, indicated values of *V*_*d*_ are those applied *directly* to the graphene region under study. These values are determined by utilising the small-signal AC measurements as a calibration; with a knowledge of *v*_*d*_, the external-circuit components, and our measurement of the actual AC voltage drop (*v*) across the graphene channel, we define a ratio (*v*/*v*_*d*_) that we use to determine the actual DC voltage drop across the channel (*V*_*d*_ = *V*_*d*_^*appl*^ × (*v*/*v*_*d*_)). In this expression, *V*_*d*_^*appl*^ is the DC voltage applied to the outer contacts of the device. Finally, to suppress the influence of quantum corrections, the differential conductance or resistance (*g*_*d*_^−1^) was measured by applying a small, but fixed, value of *V*_*d*_ and subsequently determining the variation of *g*_*d*_(*T*)|_*Vd*_ or *g*_*d*_^−1^(*T*)|_*Vd*_.

## Electronic supplementary material

Evaluating the Sources of Graphene’s Resistivity Using Differential Conductance
