# Supplementary material for: Evaluating the Sources of Graphene’s Resistivity Using Differential Conductance
Source: Sci Rep. 2017 Sep 4;7:10317. doi: 10.1038/s41598-017-10367-1 (PMC5583290; doi:10.1038/s41598-017-10367-1)
Supplement: Supplementary file 1 — Evaluating the Sources of Graphene’s Resistivity Using Differential Conductance [file 41598_2017_10367_MOESM1_ESM.pdf]

SUPPLEMENTARY INFORMATION

Evaluating the Sources of Graphene's Resistivity Using Differential Conductance

R. Somphonsane[1,*], H. Ramamoorthy[2], G. He[2], J. Nathawat[2], C.-P. Kwan[3],

N. Arabchigavkani[3], Y.-H. Lee[2], J. Fransson[4] & J. P. Bird[2,5]

S1. Basic Device Characterization

We have investigated the basic characterization of six different graphene devices, two of which were monolayer and four of which were bilayer. Table S1 summarizes the important parameters for these devices, with the notation "M" and "B" referring to monolayer and bilayer graphene, respectively. M2 and B4 are the devices investigated in the main paper.

Device	L [a] (μm)	W [a] (μm)	μ_h [b] (cm^2/Vs)	μ_e [b] (cm^2/Vs)	σ_{min} [c] (e^2/h)
M1	0.8	0.4	16500	10200	23
M2	1.1	1.3	14200	11600	22
B1	1	4	1100	1070	4.2
B2	4	0.25	1100	1280	4
B3	1.5	1	2300	1800	5.5
B4	1	2	420	450	1.3

Table S1. Important parameters for the different devices studied. [a]L, W: channel length, width, measured between voltage probes. [b]μ_e, μ_h: Electron, hole mobility, determined at 4 K, and at density of 10^{12} cm^{-2}. [c]σ_{min}: minimum conductivity, measured at 4 K.

From the Table above it can be seen that the mobility values exhibited by the different types of device are consistent; the monolayer devices show much higher mobility than the bilayer ones, but, for

a given device type, the observed mobility values are similar. This is in spite of the different contact geometries employed in the different devices.

S2. Raman Spectroscopy of Monolayer and Bilayer Graphene

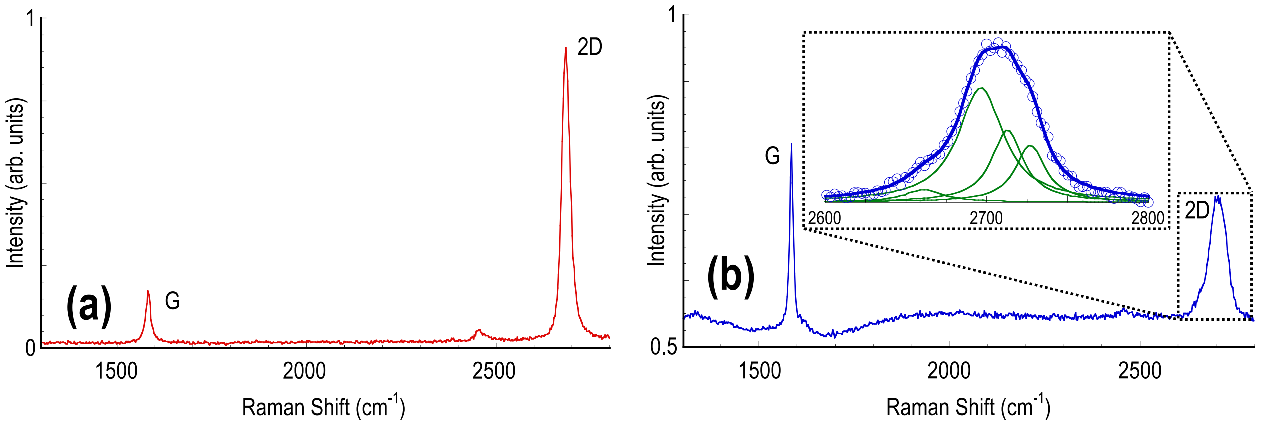

Figure S1. The Raman spectrum of the (a) monolayer device (M2), and (b) bilayer device (B4), measured under ambient conditions. The inset presents an expanded view of the 2D band near 2700 cm^{-1}, indicating how it may be fit in terms of the contribution from four independent Lorentzians (green lines). The sum of these four peaks is denoted by the blue line, while the experimental data are plotted here as empty symbols.

Layer identification of the above devices was achieved through a combination of optical microscopy and Raman imaging. Raman spectra of these devices were measured at room temperature under an excitation wavelength of 514 nm. Raman spectra of the monolayer and bilayer devices studied in the main paper (M2 & B4) are shown in Fig. S1. Panel (a) shows the spectrum of device M2 (monolayer), with the two most prominent features being the G peak at 1582 cm^{-1} and the 2D band at around 2700 cm^{-1}. The number of graphene layers is determined from the shape of the 2D band, and from the ratio of the intensity of the G peak and the 2D band[1-3] (I(G)/I(2D)). Here we measure a sharp 2D peak with an intensity ratio I(G)/I(2D) of ~0.17, consistent with previous studies of monolayer graphene.[1-3] The Raman spectrum of the bilayer device (B4) is shown in panel (b). The 2D band here is much broader than

that in the monolayer, and is also up-shifted with respect to that material. A Lorentzian fitting of the 2D peak yields good agreement by including four distinct peaks (see the inset to Fig. S1(b)), confirming the bilayer nature of the graphene.[1-3] The intensity ratio I(G)/I(2D) of about 1.1 is also consistent with prior studies of bilayer devices.

S3. Linear Conductance of Monolayer and Bilayer Graphene

Here we describe the linear conductance measurements of the devices mentioned in Table S1, which consist of measuring their source-drain conductance as a function of back-gate voltage (i.e. their Dirac curves). These measurements were made at a series of different temperatures (while sourcing a fixed current of 100 nA). In Figs. S2 – S3, we show the comparison of these different measurements for the different devices mentioned above.

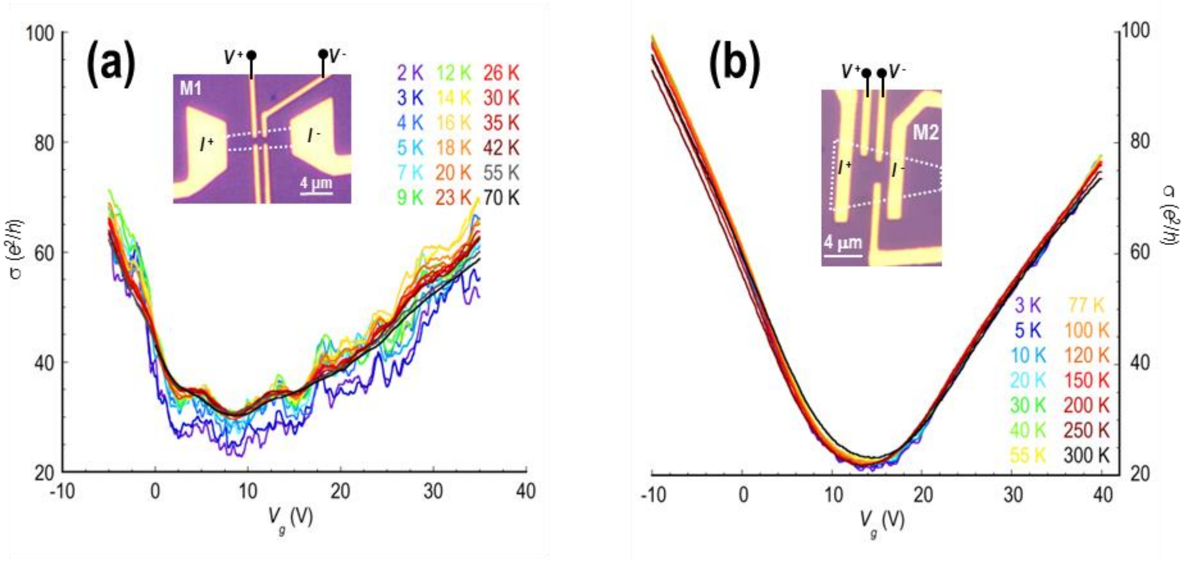

Figure S2. Dirac curves at various temperatures for device M1 (a) and M2 (b). The Dirac point can be clearly identified from the high-temperature curves in each panel and occurs at approximately 8 V for device M1 and 14 V for M2. Optical micrographs of these devices are shown in the corresponding insets, in both of which the outline of the graphene flake is indicated by the white dotted line and current and voltage probes are indicated.

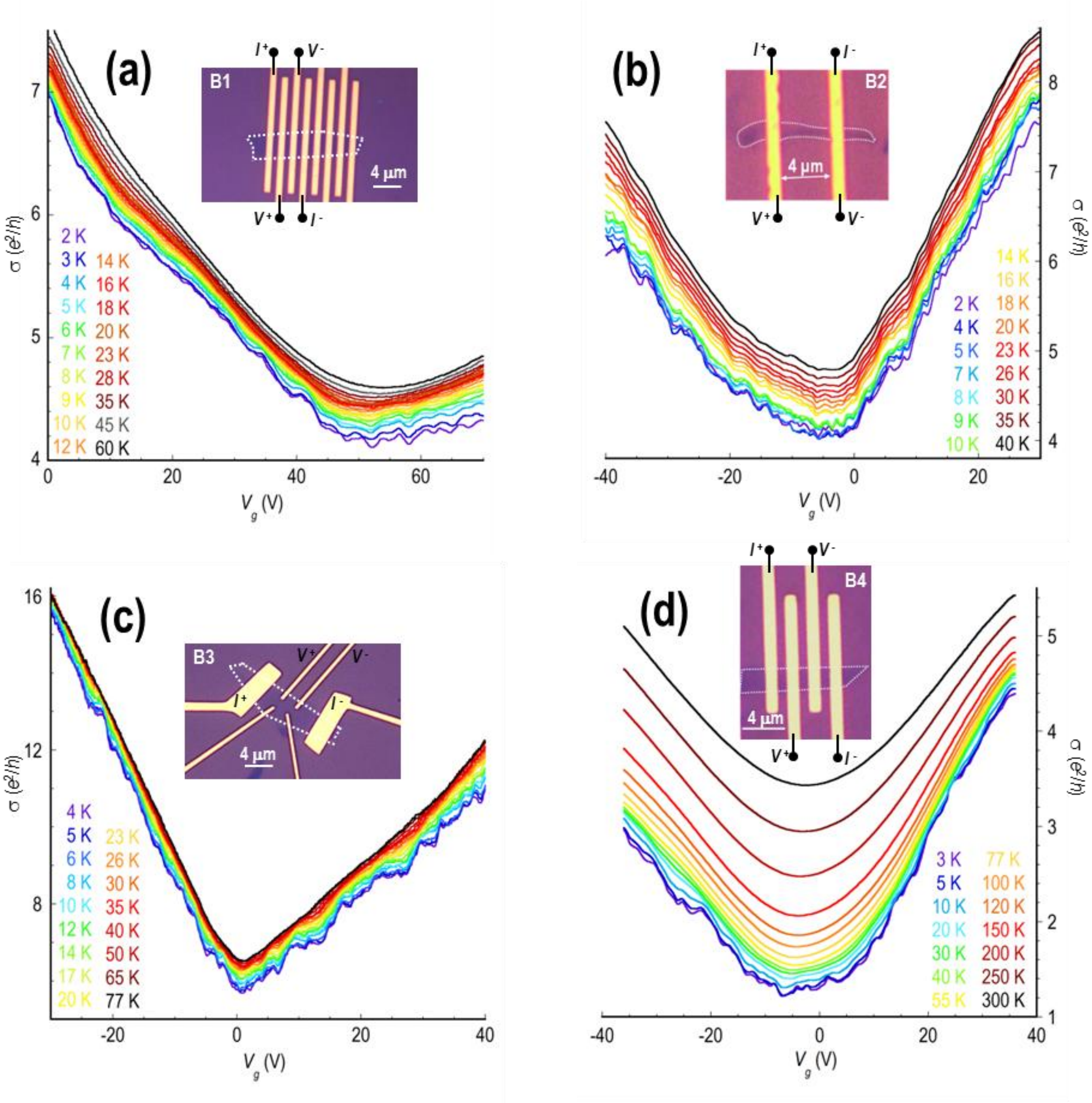

Figure S3. Dirac curves at various temperatures for device B1 (a), B2 (b), B3 (c), and B4 (d). The Dirac point can be clearly identified from the high-temperature curves in each panel and occurs at approximately 55 V for device B1, -5 V for B2, 0 V for B3 and -5 V for B4. Optical micrographs of these devices are shown in the corresponding insets, in both of which the outline of the graphene flake is indicated by the white dotted line and current and voltage probes are indicated.

With regards to the similarities between the two monolayer devices in Fig. S2, it will be apparent that mesoscopic effects are much stronger in Device M1 than in M2, which may be due to the small-

er voltage-probe separation in M1. Nonetheless, the mesoscopic features are strongly suppressed in M1 once the temperature is increased to ~20 K, above which the temperature-dependent variations of the conductance in Fig. S2(a) resemble those in Fig. S2(b) (although it should be noted that the variation of conductance with temperature is measured over a wider range in Fig. S2(b)).

S4. Carrier-Concentration Analysis

In any analysis of the conductivity mechanisms in graphene, it is necessary to recognize that the conductivity (or resistivity) is determined by the combined influence of two parameters, namely the carrier concentration and the mobility. In graphene, the electron (n) and hole (p) concentrations include contributions from[4] gate-induced (n_g) and thermally-generated (n_{th}) carriers, as well as from the residual puddle density (n^*):

$$n, p \approx \frac{1}{2}\left[\pm\left(n_g + \sqrt{n_g^2 + 4n_0^2}\right)\right], \qquad \text{(S1)}$$

where the minus (plus) sign corresponds to electrons (holes). Here, $n_g = -C_{ox}V_{g_0}/q$, where $C_{ox} = \varepsilon_{ox}/t_{ox}$ is the gate capacitance per unit area, $\varepsilon_{ox} = 3.9$ is the dielectric constant of SiO_2, q is the elementary charge, and $V_{g_0} = V_g - V_0$ is the gate voltage referenced to the Dirac point. The values for n_g calculated for the monolayer and bilayer devices studied in the main paper are shown in the tables below. The residual and thermal concentrations are taken into account through the minimum carrier density $n_0 = [(n^*/2)^2 + n_{th}^2]^{1/2}$, where $n_{th} = (\pi/6)(k_BT/\hbar v_F)^2$ in monolayer graphene (with v_F the Fermi velocity) and $n_{th} = \ln2(2m^*/\pi\hbar^2)k_BT$ in bilayer[5] (with $m^* = 0.033m_0$ being the electron or hole effective mass).

V_g (V)	14	12	10	8	6	4	2	-2	-6	-10
n_g (10^{12} cm^{-2})	0	0.14	0.29	0.43	0.58	0.72	0.86	1.2	1.4	1.7

Table S2. Values of n_g at different values of V_g for the monolayer device (M2). The Dirac point in this device is located at V_{g_0} = 14 V.

V_g (V)	-6	-2	2	6	12	18	24	30	36
n_g (10^{12} cm^{-2})	0.13	0.16	0.45	0.73	1.2	1.6	2.0	2.5	2.9

Table S3. Values of n_g at different values of V_g for the bilayer graphene (B4). The Dirac point in this device is located at V_{g_0} = -5 V.

In Fig. S5, we use Eq. (S1) to analyse the influence of residual and thermal carriers on the total charge concentration in monolayer (Fig. S3(a)) and bilayer (Fig. S3(b)) graphene. The graphs actually plot the variation of the total carrier concentration (n or p) as a function of temperature, along with the corresponding contributions from gate-induced (n_g) and residual and thermal (n_0) carriers. To obtain the variations shown in Fig. S3, n^* was determined[6,7] from the width of the minimum-conductivity plateau in the transconductivity ($\sigma(V_g)$, in units of e^2/h per square), as demonstrated in Fig. S4. At a reference temperature of 40 K, n^* was thus found to be 3.5×10^{11} cm^{-2} for the monolayer device (with a plateau width of ~ 4.8 V, and a minimum conductivity σ_0 ~$22e^2/h$, see Fig. S4(a)) and 7.7×10^{11} cm^{-2} for bilayer graphene (with a plateau width of ~ 10 V and a minimum conductivity σ_0 ~$1.6e^2/h$, see Fig. S4(b)). By making use of the self-consistent theory discussed in Refs. 6 & 7, the concentration of charged impurities responsible for such residual carriers could then be calculated to be around 3.1×10^{11} cm^{-2} and 9.3×10^{11} cm^{-2} for the monolayer and bilayer devices, respectively. These values are fairly common for graphene devices formed on SiO_2,[6,7] and are consistent also with the charged-impurity concentration that we infer from the intensity ratio of the Raman peaks,[8,9] I(G)/I(2D).

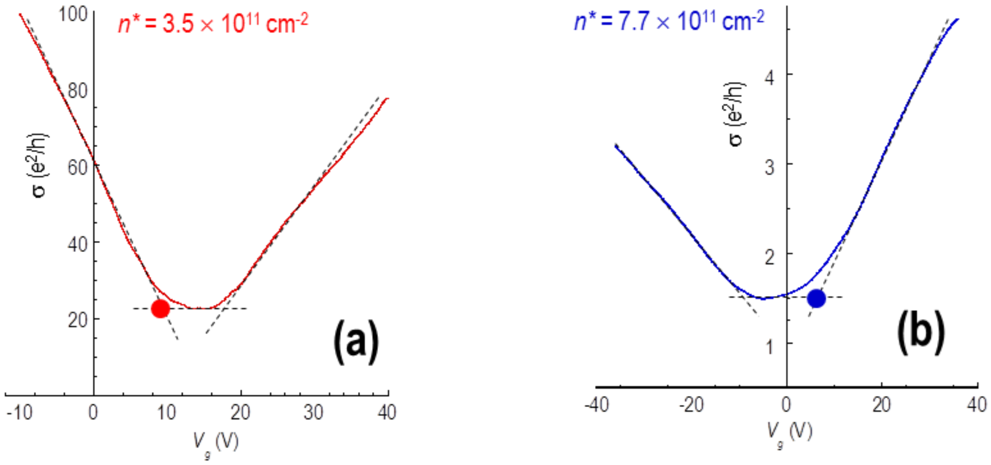

Figure S4. The method used to infer the residual charged density (n^*) from the transfer curve at 40 K of the monolayer (a) and (b) bilayer (b) device. Corresponding values of n^* are indicated in each panel.

Turning now to the results of Fig. S5, in Fig. S5(a) we indicate the temperature dependence of the hole density (p) in the monolayer device, for different characteristic gating conditions. (A similar analysis could have been performed for electrons in the same device, without significantly changing any of our conclusions). In each of these panels, we plot the variation of $n_0(T)$ by experimentally determining the residual carrier density at each temperature, and then combining this with the calculated thermal-carrier concentration. In the top panel, the gate-induced component of the density is relatively large ($\sim 1.7 \times 10^{12}$) and consequently dominates the total concentration at all temperatures. In the bottom panel, in contrast, the device is configured near the Dirac point and the carrier concentration is due entirely to the residual and thermal carriers. In the remaining panels of the figure, the density is adjusted between these two limits, allowing one to appreciate how the residual and thermal concentrations become increasingly important as the Dirac point is approached. An additional feature revealed by each of the panels of Fig. S5(a) is that the minimum concentration n_0 does not show any significant dependence of temperature. This is due to the fact that residual carriers dominate over the thermal ones in monolayer graphene ($n^* > n_{th}$), at all temperatures considered here.

Turning next to the results of Fig. S5(b), the four panels of this figure show the results of a carrier-concentration analysis for the bilayer device. Here we are concerned with gate induced variations in

the electron concentration, although, again, this analysis could just as equally have been performed for holes. The different panels of this figure follow a similar sequence to Fig. S5(a), and roughly show analogous behaviour; when the gate-induced concentration is high the role of thermal and residual carriers is minimal, whereas as the Dirac point is neared the latter carriers dominate. A significant difference between these results and those of Fig. S5(a), however, is that the role of thermal carriers is more prominent in the bilayer than in the monolayer system. Specifically, it is apparent in Fig. S53(b) that n_0 roughly doubles as the temperature is increased to 300 K, a change that can be attributed almost exclusively to a corresponding increase in $n_{th}(T)$. In the discussion that follows, it will therefore be particularly necessary to pay attention to the influence of thermal carriers when discussing the temperature-dependent conductivity variations in the bilayer system.

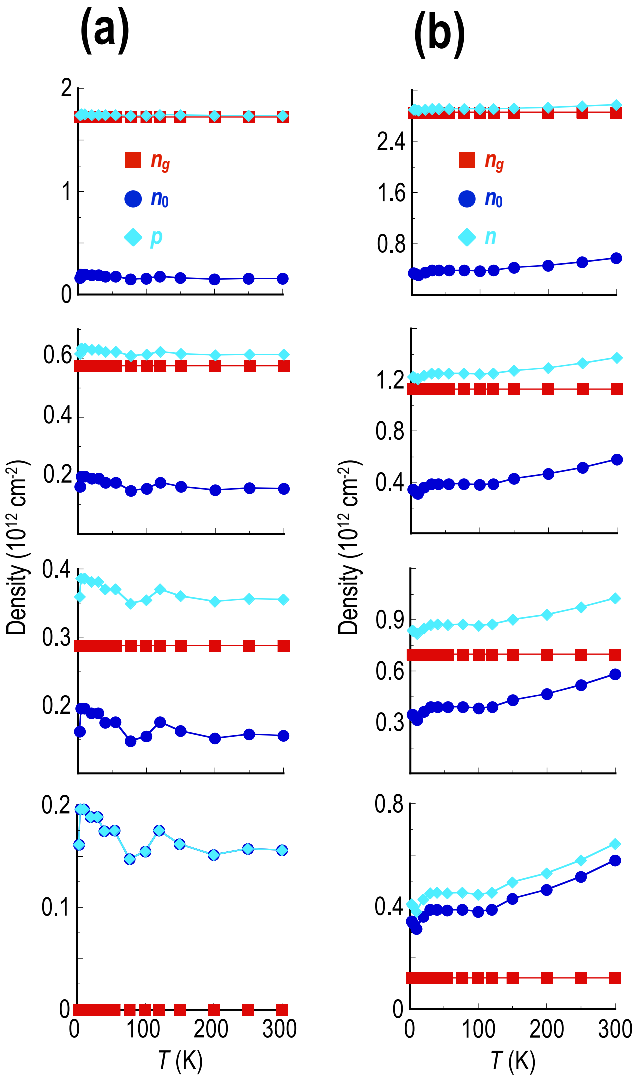

Figure S5. (a) Contributions to the total hole density in the monolayer device at various temperatures from 3 – 300 K. The four different panels correspond to four-different values of the gate-induced hole concentration (n_g) and the minimum carrier density (n_0) was determined from the residual charge density (n^*) inferred from the transfer curves (see Fig. S4) and from the computed thermal concentrations (n_{th}). Moving from the top panel to the bottom one sequentially, the gate voltages (V_g) were -10 V, 6 V, 10 V, and 14 V. (b) Corresponding contributions to the total electron density in the bilayer device at various temperatures from 3 – 300 K. Gate voltages from top to bottom are V_g = 36 V, 12 V, 6 V, and -2 V.

S5. Analysis of Quantum Corrections Based on Magneto-Conductance Measurements

The zero-bias peak seen in differential resistance g_d^{-1} at low temperatures can be attributed to quantum corrections, most notably to the combined influence of weak localization [10-13] and electron interactions [14,15]. A well-known approach to probe these phenomena involves the application of a perpendicular magnetic field, which breaks time reversal symmetry and suppresses weak localization, while leaving electron interactions largely unaffected [16]. Motivated by this, in Fig. S6(a) we plot the low-temperature (3-K) magneto-conductance of the monolayer device (at V_d = 0). The first feature we note is the asymmetric character of the magneto-conductance with respect to field reversal, which reflects the fact that our four-terminal geometry does not yield a perfect longitudinal measurement but also contains an admixture of a Hall component. Consequently, in Fig. S6(b) & S6(c) we reconstruct the magneto-conductance in terms of symmetric and antisymmetric components. To do this the magneto-resistance at positive and negative magnetic field was either mirrored symmetrically ($G_{sym}(\pm B)$= ($G(+B)$ + $G(-B)$)/2) or antisymmetrically ($G_{asym}(\pm B)$= $\pm$($G(+B)$ - $G(-B)$)/2) around zero field. We found that the anti-symmetric component does indeed correspond very well to a linear Hall term. More importantly, the symmetrized component of the magneto-conductance clearly shows a region of positive magneto-conductance that is centered around zero field. The full width of this region is ~50 mT, consistent with previous reports of negative magneto-resistance due to weak localization in graphene [11,13]. Indeed, in Fig. S6(b) we see that this negative magneto-resistance is suppressed with increase of temperature to ~50 K, where the reproducible magneto-fluctuations that also arise from quantum interference [17,18] are similarly strongly damped. This temperature scale correlates well to that for which the zero-bias peak in differential resistance is suppressed (see Fig. 2 of the main paper). While these results suggest a strong connection of the zero-bias peak to weak localization, this phenomenon alone is, as mentioned in the main paper, insufficient to account for its full amplitude. To see this point cons*i*der the inset of Fig. 1(a) in the main paper, where we plot measurements of differential conductance as a function of V_d, for three static values of the magnetic field. The largest of these (0.4 T) is around an order of magnitude larger than that required to quench weak localization, yet we see that the zero-bias peak persists, albeit with an amplitude that is reduced by ~50% compared to that at zero magnetic field. From this observa-

tion, we infer that the zero-bias peak seen in differential resistance reflects a combination of weak localization and electron interactions, both of which become increasingly important as the temperature is lowered and the phase coherence of the carriers is enhanced [19]. It is well known from the study of conventional metals and semiconductors that, in the presence of weak disorder, the classical (Boltzmann) conductivity exhibits corrections, not only due to weak localization, but also due to electron-electron interactions [19-21]. The interactions influence transport well into the metallic limit ($k_F l \gg 1$) [19-21], and have also been reported for graphene [22]. In terms of the connection of this magneto-resistance feature to the peak in differential resistance, when $|V_d|$ is increased from zero in this regime the ensuing increase in the dephasing rate is then presumably responsible for the drop in differential resistance that is seen in Figs. 2 & 3 of the main paper.

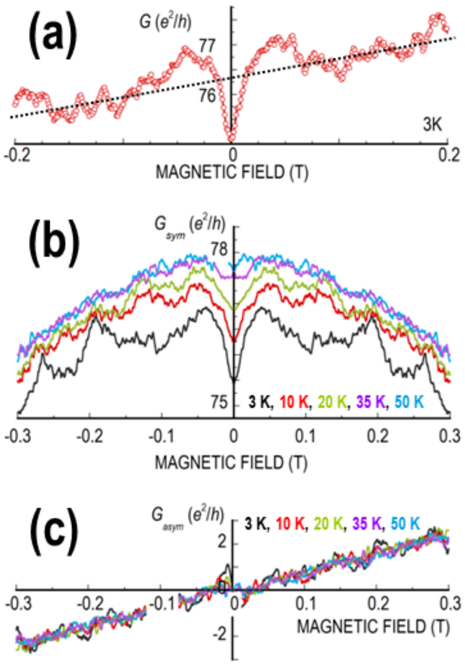

Figure S6. (a) The linear magneto-conductance (V_d = 0) measured at the lowest temperature (3 K) for the monolayer device. The black dotted line denotes a linear dependence on magnetic field, indicative of a Hall component. (b) Symmetric and (c) antisymmetric components of the linear magneto-conductance at various temperatures in the range of 3 – 50 K. The zero-magnetic-field conductance dip is washed away at ~50 K, which correlates closely to the temperature scale associated with the quenching of the quench zero-bias peak in differential resistance in Fig. 2 of the main paper.

S6. Results for Additional Bilayer Device

In addition to our studies of the linear conductance presented in Section S3, we have also performed an detailed investigation of the differential conductance of an additional bilayer device (B2), allowing us to establish the generality of the behavior exhibited by the device (B4) considered in the main paper. In Fig. S7, we plot the temperature dependence of the resistance determined for three different gate voltages for this device. The Dirac point in this device occurs at V_g = -5 V, corresponding to the center panel in Fig. S7, while the the left and right panels of this figure show the resistance variations for hole and electron transport, respectively. In all panels, data are plotted for multiple values of the DC bias (V_d) and it is clear that the resulting variations are consistent with those reported in Fig. 4 of the main paper. In fact, we note that a similar insulating behavior for the resistance has also been discussed in an independent study of bilayer graphene transistors [23], although in that work only a narrow range of temperature near 300 K was explored and the microscopic origins of the observed behavior was not considered.

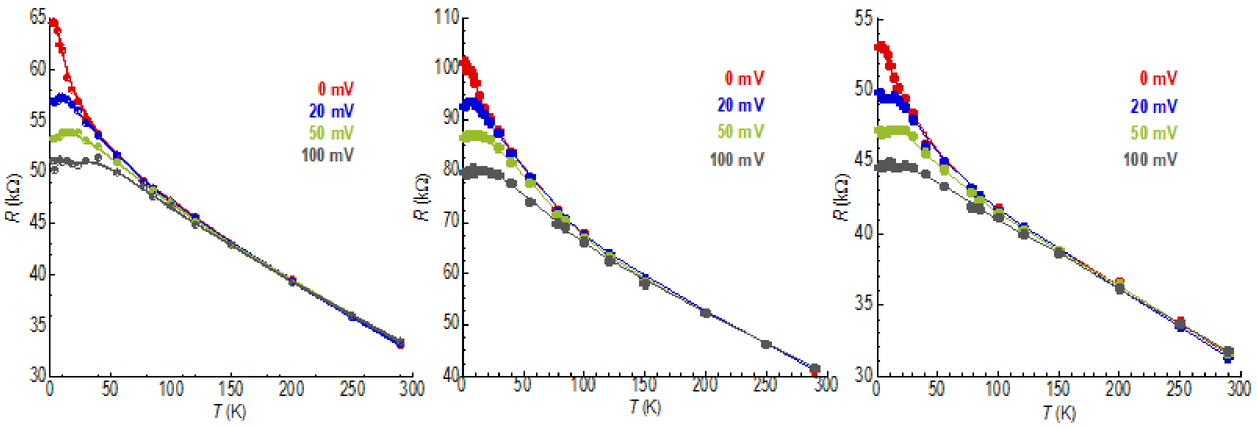

Figure S7. Temperature dependent variation of the resistance in bilayer device B2. The centre panel corresponds to the Dirac point, so that the left and right panels of this figure show the resistance variations for hole and electron transport, respectively. In each panel, the variation of resistance with temperature is plotted for several fixed values of V_d.

References

1. Ferrari, A. C. *et al*. Raman Spectrum of Graphene and Graphene Layers. *Phys. Rev. Lett.* **97**, 187401 (2006).
2. Gupta A., Chen, G. Joshi, P., Tadigadapa, S. & Eklund, P. C. Raman scattering from high-frequency phonons in supported n-graphene layer films. *Nano Lett.* **6**, 2667–2673 (2006).
3. Graf. D. *et al*. Spatially Resolved Raman Spectroscopy of Single- and Few-Layer Graphene. *Nano Lett.* **7**, 238–242 (2007).
4. Dorgan, V. E., Bae, M.-H. & Pop, E. Mobility and saturation velocity in graphene on SiO_2. *Appl. Phys. Lett.* **97**, 082112 (2010).
5. Bae, M.-H., Ong, Z.-Y., Estrada, D. & Pop, E. Imaging, Simulation, and Electrostatic Control of Power Dissipation in Graphene Devices. *Nano Lett.* **10**, 4787 (2010).
6. Adam, S., Hwang, E. H., Galitski, V. M. & Das Sarma, S. A self-consistent theory for graphene transport. *Proc. Natl. Acad. Sci. U.S.A.* **104**, 18392 (2007).
7. Adam, S. & Das Sarma, S. Boltzmann transport and residual conductivity in bilayer graphene. *Phys. Rev. B* **77**, 115436 (2008).
8. Casiraghi, C., Pisana, S., Novoselov, K. S., Geim, A. K. & Ferrari, A. C. Raman Fingerprint of Charged Impurities in Graphene. *Appl. Phys. Lett.* **91**, 233108 (2007).
9. Ni, Z. H. *et al*. Probing Charged Impurities in Suspended Graphene Using Raman Spectroscopy. *ACS Nano* **3**, 569–574 (2009).
10. McCann, E. *et al*. Weak-Localization Magnetoresistance and Valley Symmetry in Graphene. *Phys. Rev. Lett.* **97**, 146805 (2006).
11. Wu, X.; Li, X.; Song, Z.; Berger, C.; de Heer, W. A. Weak Antilocalization in Epitaxial Graphene: Evidence for Chiral Electrons. *Phys. Rev. Lett.* **98**, 136801 (2007).
12. Gorbachev, R.V.; Tikhonenko, F.V.; Mayorov, A. S.; Horsell, D. W.; Savchenko, A. K. Weak Localization in Bilayer Graphene, *Phys. Rev. Lett.* **98**, 176805 (2007).

13. Tikhonenko, F. V.; Horsell, D. W.; Gorbachev, R. V.; Savchenko, A. K. Weak Localization in Graphene Flakes. *Phys. Rev. Lett.* **100**, 056802 (2008).

14. Kozikov, A. A.; Savchenko, A. K.; Narozhny, B. N.; Shytov, A. V. Electron-Electron Interactions in the Conductivity of Graphene. *Phys. Rev. B* **82**, 075424 (2010).

15. Jouault, B. *et al*. Interplay Between Interferences and Electron-Electron Interactions in Epitaxial Graphene. *Phys. Rev. B* **83**, 195417 (2011).

16. Choi, K. K.; Tsui, D. C.; Palmateer, S. C. Electron-Electron Interactions in GaAs-$Al_xGa_{1-x}As$ Hetero-structures. *Phys. Rev. B* **33**, 8216 (1986).

17. Bohra, G. *et al*. Nonergodicity and Microscopic Symmetry Breaking of the Conductance Fluctuations in Disordered Mesoscopic Graphene. *Phys. Rev. B* **86**, 161405(R) (2012).

18. Bohra, G. *et al*. Robust Mesoscopic Fluctuations in Disordered Graphene. *Appl. Phys. Lett*. **101**, 093110 (2012).

19. Lin, J. J.; Bird, J. P. Recent Experimental Studies of Electron Dephasing in Metal and Semiconductor Mesoscopic Structures. *J. Phys.: Cond. Matt*. **14**, R501 – R596 (2002).

20. Choi, K. K.; Tsui, D. C.; Palmateer, S. C. Size effects on electron-electron interactions in GaAs-$Al_xGa_{1-x}As$ heterostructures. *Phys. Rev. B* **32**, 5540(R) (1985).

21. Choi, K. K.; Tsui, D. C.; Palmateer, S. C. Electron-electron interactions in GaAs-$Al_xGa_{1-x}As$ hetero-structures. *Phys. Rev. B* **33**, 8216 (1986).

22. Kozikov, A. A., Savchenko, A. K., Narozhny, B. N. & Shytov, A. V. Electron-Electron Interactions in the Conductivity of Graphene. *Phys. Rev. B* **82**, 075424 (2010).

23. Mahjoub, A. M., Suzuki, S., Ouchi, T., Aoki, N., Miyamoto, K., Yamaguchi, T., Omatsu, T., Ishibashi, K. & Ochiai, Y. Terahertz bolometric detection by thermal noise in graphene field effect transistor. *Appl. Phys. Lett*. **107**, 083506 (2015).
